# Supplementary material for: Correlation of fitness landscapes from three orthologous TIM barrels originates from sequence and structure constraints
Source: Nat Commun. 2017 Mar 6;8:14614. doi: 10.1038/ncomms14614 (PMC5343507; doi:10.1038/ncomms14614)
Supplement: Supplementary Information — Supplementary Figures and Supplementary Tables [file ncomms14614-s1.pdf]

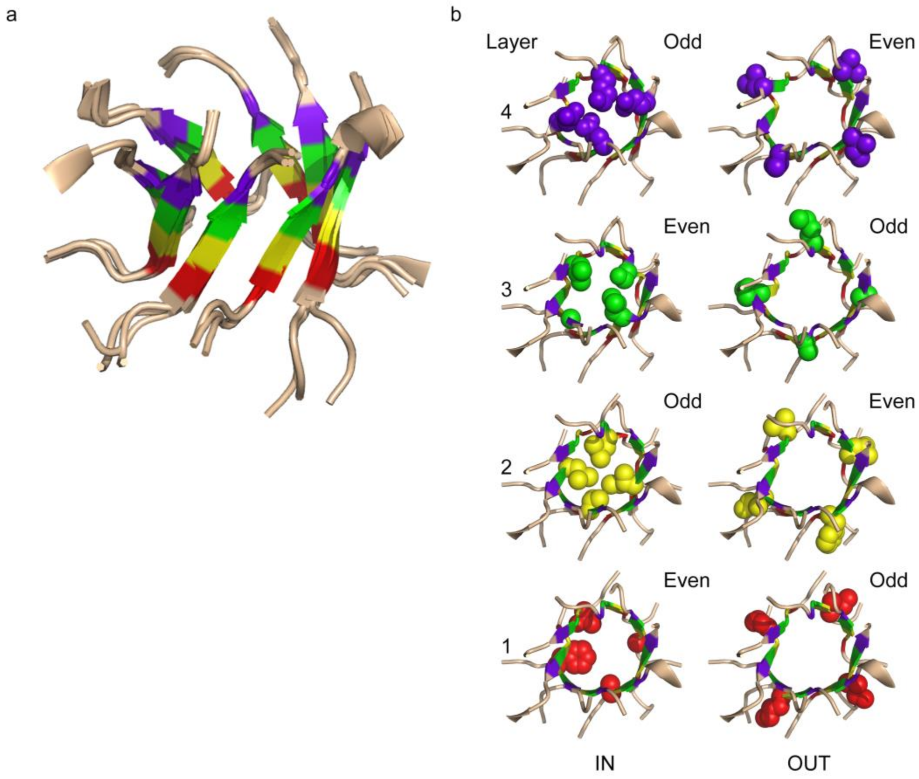

Supplementary Figure 1. Canonical layers of the β-barrel stabilize the protein core and provide surface area for docking the α-helices. (a) Four layers of the β-strands forming the TIM barrel core are colored red, yellow, green, and purple, from the N-terminus to C-terminus. (b) Orientation of side chains alternate in and out of the β-barrel per strand and per layer to maximize packing and to reduce steric clashes. Alternating side chain orientation evenly distributes stabilizing hydrophobic interactions within the β-barrel core and between the β-strands and surrounding α-helices.

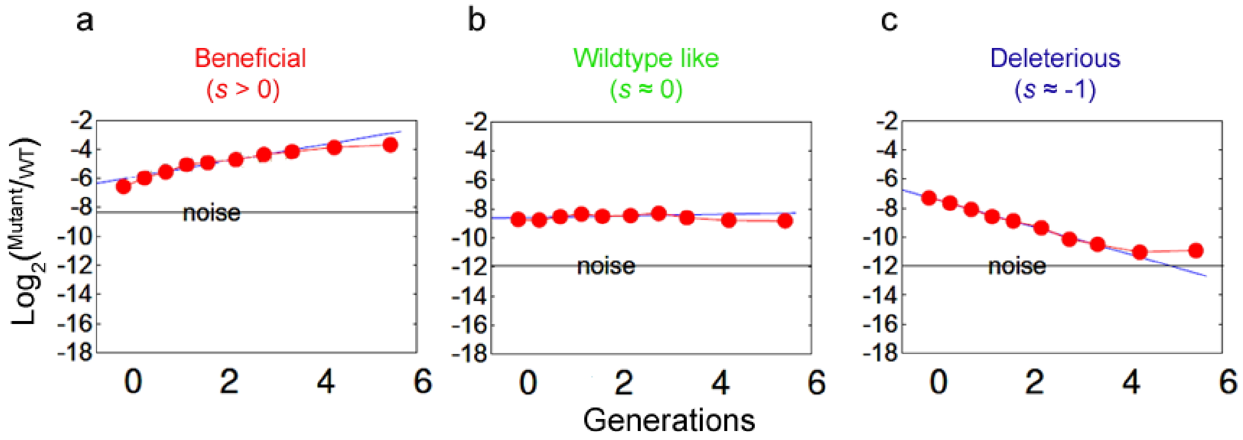

Supplementary Figure 2. Selection coefficient is determined by the slope of the relative abundance of mutant to WT IGPS over time. Representative examples of the three observed fitness phenotypes are displayed on a $\log_2$ scale to show doubling time. (a) Beneficial mutations have selection coefficient greater than 0, indicated by the positive slope of the relative abundance of mutant to WT over time. (b) WT-like mutations have selection coefficient approximately 0, indicated by the flat character of the relative abundance of mutant to WT over time. (c) Deleterious mutations have selection coefficient of approximately -1, indicated by the negative slope of the relative abundance of mutant to WT over time. The noise level line indicates the abundance of the mutant sequence obtained from deep sequencing of a WT sample.

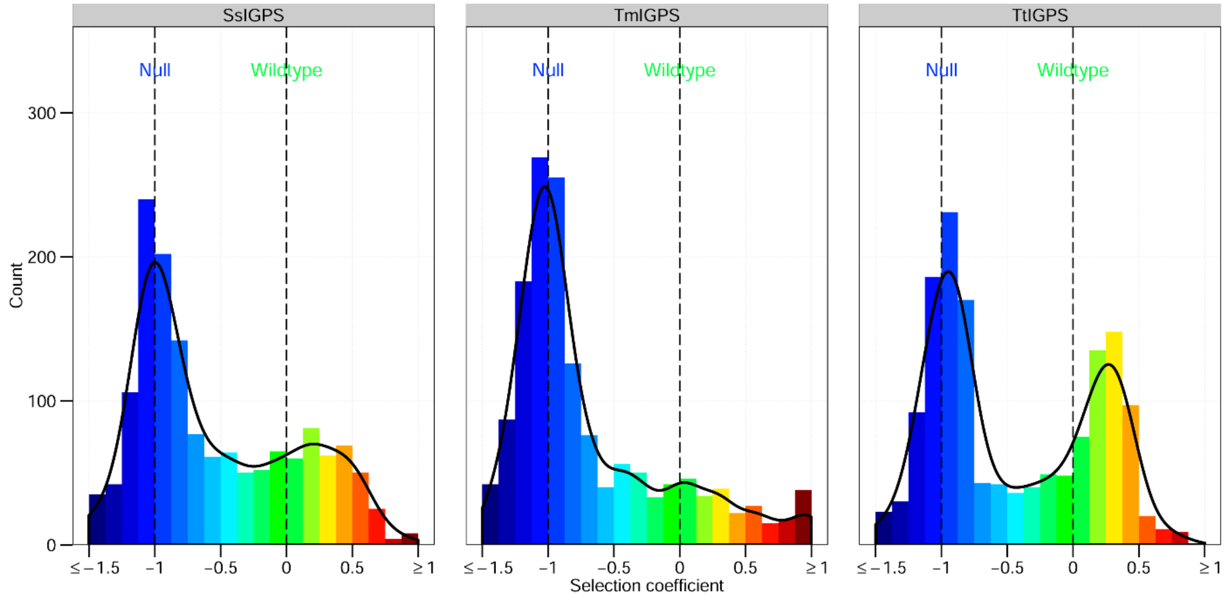

Supplementary Figure 3. Distribution of fitness values for three orthologous IGPS proteins. Distribution of fitness values for SsIGPS (left), TmIGPS (center), and TtIGPS (left) plotted on a histogram. Bimodal distributions were observed for all three orthologs, centered at s = -1 and centered above s = 0. Fitness values above 0 indicate a fitness gain due to mutation.

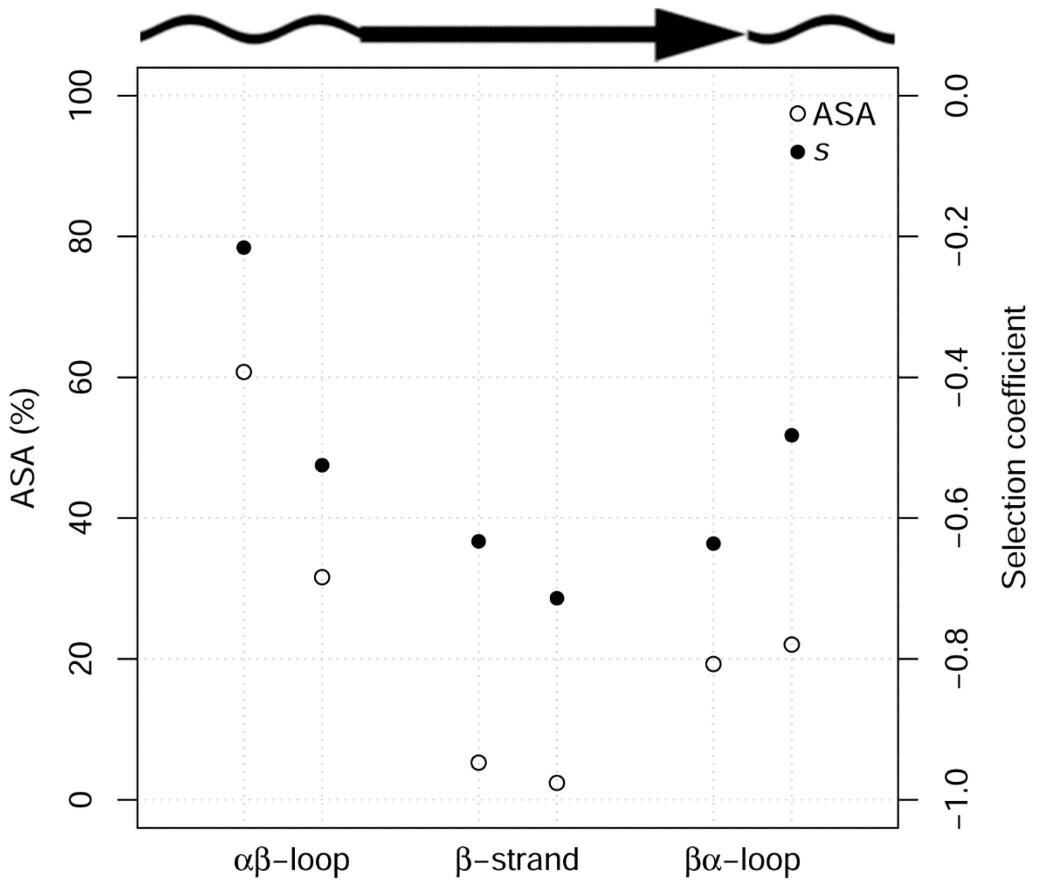

Supplementary Figure 4. Accessible surface area and fitness vary by secondary structure and strand parity. Average fitness varied depending on both secondary structure and strand parity (● Selection coefficient, right axis). Average fitness correlates to ASA of these stratified groups (○ ASA, left axis).

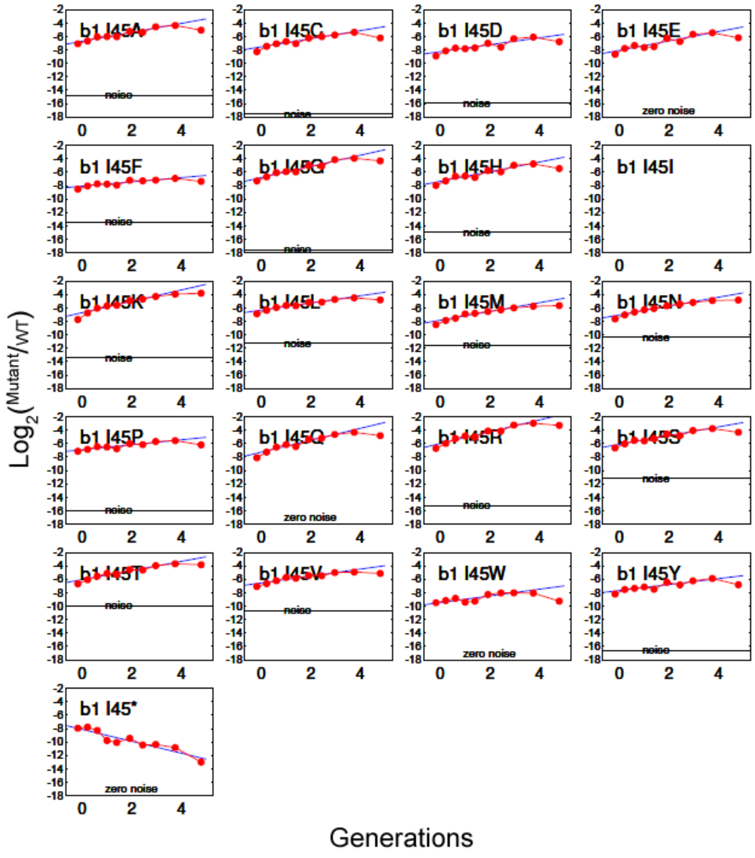

Supplementary Figure 5. The effect of mutations at SsIGPS I45 on fitness.

Mutations to SsIGPS I45 were uniformly beneficial, except for mutation to the stop codon. Tryptophan biosynthesis is a highly regulated process, where tryptophan accumulation leads to feedback inhibition at the first step of synthesis [Braus, G. H. Aromatic amino acid biosynthesis in the yeast Saccharomyces cerevisiae: a model system for the regulation of a eukaryotic biosynthetic pathway. *Microbiol. Rev.* **55,** 349–370 (1991)]. By the fourth generation, mutant and WT abundances were comparable, suggesting that a steady state of tryptophan concentration has been reached.

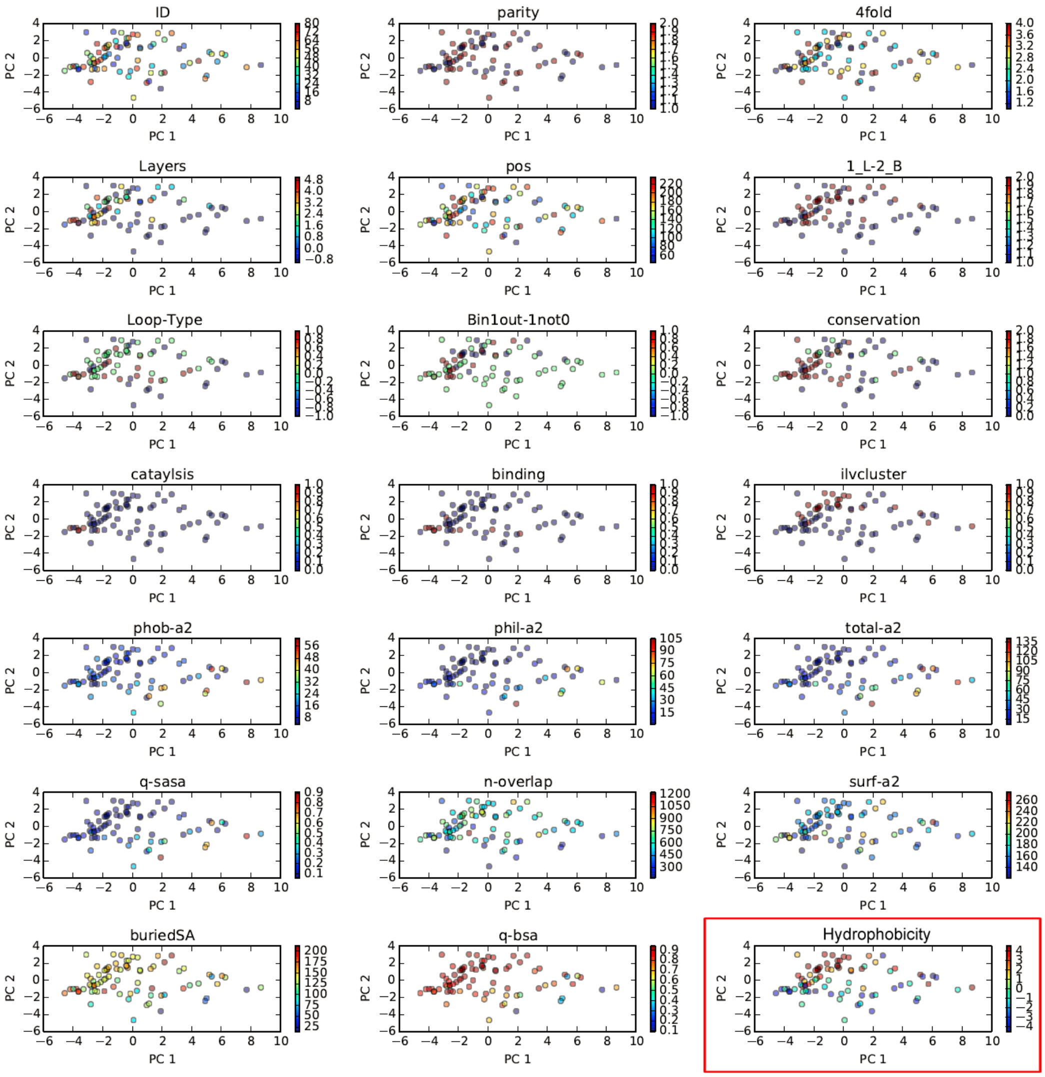

Supplementary Figure 6. Representative biplot of the secondary principal component (PC2) *vs*. the first principal component (PC1) of the PCA for SsIGPS. Several biochemical and structural features were examined to identify major sources of fitness variance in our EMPIRIC dataset. Biplots did not reveal a direct relationship between PC1 and the features examined. Hydrophobicity based on the Kyte-Doolittle scale was identified as the second largest factor influencing fitness, indicated by the monotonic color change along the PC2 axis (last plot highlighted by the red box).

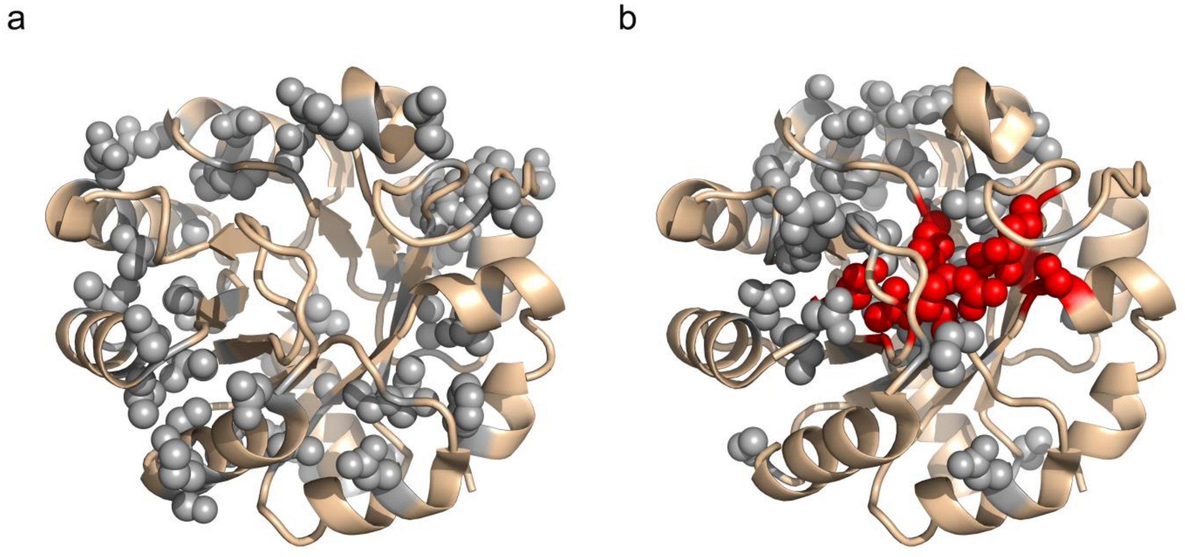

Supplementary Figure 7. SCA sectors in IGPS TIM barrel proteins represented on SsIGPS. (a) Residues in sector one are highlighted with gray spheres. These residues are involved mainly in the β-strand/α-helical interface and α-helical/α-helical interface, required for stabilizing the tertiary structure. (b) Residues in sector two are highlighted with gray and red spheres. These residues are involved in protein function and stability. Residues highlighted in red are active site residues. All other residues in sector two are colored gray.

Top view **Side view**

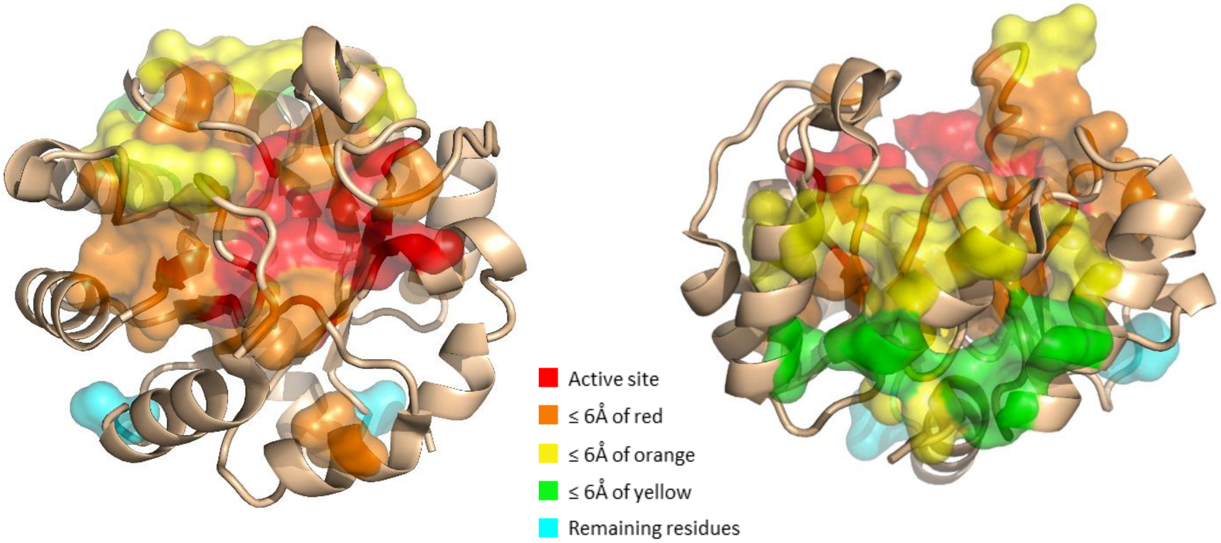

Supplementary Figure 8. Proposed conduit for allostery identified by SCA and fitness data. Sector two residues displayed by surface representation, (left) top view (right) side view, to show potential path for communication from active site to αβ-loops. Surface representation is color coded based on distance from active site residues in sector two. Active site residues are colored red. Residues within 6Å of the active site residues in sector two are colored orange. Sector 2 residues within 6Å of the orange residues are colored yellow. Sector two residues within 6Å of the yellow residues are colored green. All remaining residues in sector two are colored cyan.

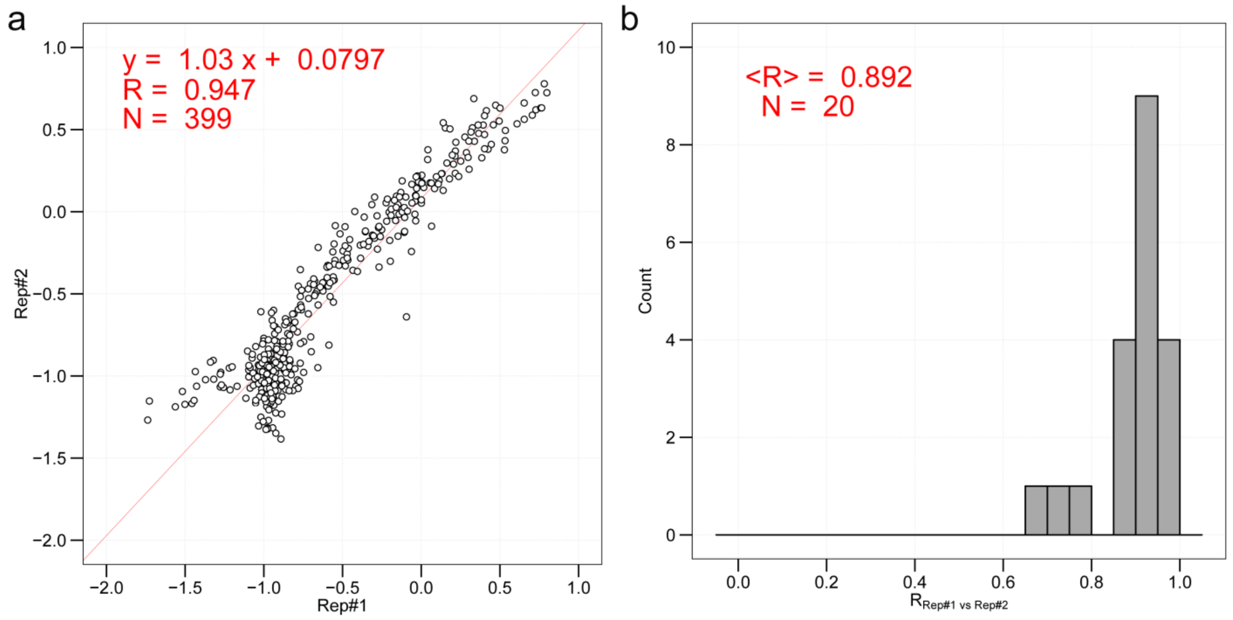

Supplementary Figure 9. Reproducibility of EMPIRIC fitness results and correlation of fitness landscapes of biological replicates of β3 and β4 libraries of SsIGPS. (a) Fitness results of full biological replicates of SsIGPS β3 and β4 are highly reproducible (R = 0.947). (b) Correlation of fitness landscape between biological replicates (20 residue positions) has a high mean R value.

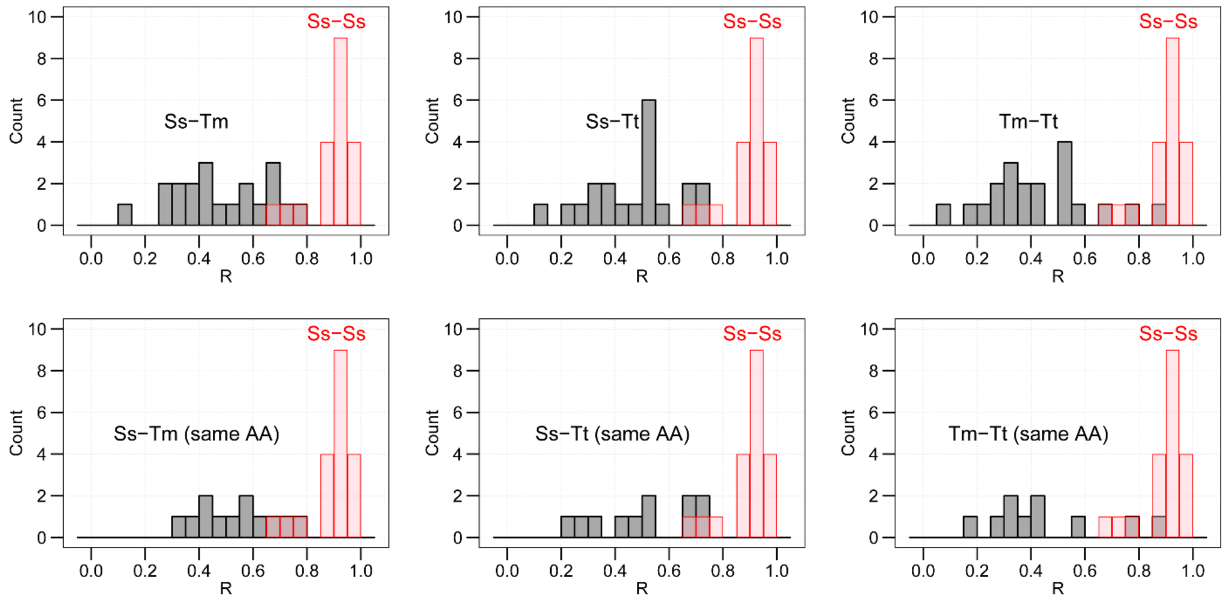

Supplementary Figure 10. Distribution of Pearson correlation *R* between fitness landscapes of SsIGPS biological replicates and of orthologs. Pearson correlation *R* between the fitness landscapes of 20 residues in SsIGPS biological replicates (red) is much stronger than the correlations between the landscapes of the orthologs (gray, all positions or positions with matching WT amino acids, $P < 10^{-4}$).

Supplementary Table 1. Comparison of correlation distributions to null distribution

P-values obtained from performing a two-sided Kolmogorov-Smirnov test comparing the distribution of correlations within specific subsets of grouped positions to the null distribution of all possible pairwise correlations.

**Identical AA**	**Count (N)**	r_{mode}	**p-value**
SsIGPS vs TmIGPS	502	0.613	< 1E-4
SsIGPS vs TtIGPS	504	0.708	< 1E-4
TmIGPS vs TtIGPS	520	0.555	< 1E-4
Null - all correlations	19200	-0.106	
**Structurally aligned positions**			
SsIGPS vs TmIGPS	78	0.621	< 1E-4
SsIGPS vs TtIGPS	78	0.724	< 1E-4
TmIGPS vs TtIGPS	80	0.617	< 1E-4
Null - all correlations	19200	-0.106	
**Structurally aligned positions, different AA**			
SsIGPS vs TmIGPS	308	0.588	< 1E-4
SsIGPS vs TtIGPS	308	0.676	< 1E-4
TmIGPS vs TtIGPS	314	0.589	< 1E-4
Null - all correlations	19200	-0.106	
**Four-fold aligned positions**			
SsIGPS vs TmIGPS	35	0.537	< 1E-4
SsIGPS vs TtIGPS	38	0.580	< 1E-4
TmIGPS vs TtIGPS	37	0.548	< 1E-4
Null - all correlations	19200	-0.106	

Supplementary Table 2. Comparison of correlation distributions between orthologs

P-values obtained from performing a two-sided Kolmogorov-Smirnov test comparing the distribution of correlations within specific subsets of grouped positions for one ortholog pair to another ortholog pair. Similar distributions are indicated by the high p-values.

**Identical AA**	**p-value**
Ss-Tm vs Ss-Tt	0.003
Ss-Tm vs Tm-Tt	0.235
Ss-Tt vs Tm-Tt	0.002
**Structurally aligned positions**	
Ss-Tm vs Ss-Tt	0.422
Ss-Tm vs Tm-Tt	0.311
Ss-Tt vs Tm-Tt	0.105
**Structurally aligned positions, different AA**	
Ss-Tm vs Ss-Tt	0.827
Ss-Tm vs Tm-Tt	0.238
Ss-Tt vs Tm-Tt	0.772
**Four-fold aligned positions**	
Ss-Tm vs Ss-Tt	0.108
Ss-Tm vs Tm-Tt	0.970
Ss-Tt vs Tm-Tt	0.126

Supplementary Table 3. Comparison of correlation distributions between subsets

P-values obtained from performing a two-sided Kolmogorov-Smirnov test comparing the distribution of correlations between specific subsets of grouped position.

Grouped subsets	p-value
Identical AA vs structurally aligned positions	<1E-4
Identical AA vs structurally aligned positions, different AA	0.668
Identical AA vs four-fold aligned positions	<1E-4
Structurally aligned positions vs structurally aligned positions, different AA	<1E-4
Structurally aligned positions vs four-fold aligned positions	<1E-4
Structurally aligned positions, different AA vs four-fold aligned positions	<1E-4

Supplementary Table 4. Amino acid composition for the four canonical β-barrel layers

Amino acid composition for the β-barrel represented in the three orthologous IGPS proteins.

	**Branched aliphatic**	**Small aliphatic**	**Aromatic**	**Acidic**	**Basic**	**Hydroxylic**	**Sulftur containing**
**In**	**20**	**11**	**1**	**9**	**3**	**4**	**0**
Layer 1	2	8	1	0	0	1	0
Layer 2	12	0	0	0	0	0	0
Layer 3	6	3	0	0	0	3	0
Layer 4	0	0	0	9	3	0	0
**Out**	**33**	**10**	**1**	**0**	**2**	**1**	**1**
Layer 1	8	2	0	0	1	1	0
Layer 2	10	1	1	0	0	0	0
Layer 3	3	7	0	0	1	0	1
Layer 4	12	0	0	0	0	0	0

The β-barrel is composed largely of branched aliphatic residues followed by small aliphatic residues. The second layer pointing into the barrel and the fourth layer pointing out of the barrel are completely composed of branched aliphatic residues, whose hydrophobic interactions stabilize the protein core and active site, respectively. The fourth layer pointing into the β-barrel is composed entirely of charged residues, where long range electrostatic interactions orient and maintain the active site, supporting catalysis.
